# Supplementary material for: Extramedullary T-lymphoblastic Crisis in a Myelodysplastic/Myeloproliferative Neoplasm with a t(12;22)/MN1::ETV6 Translocation
Source: Hematol Rep. 2023 Mar 14;15(1):212–9. doi: 10.3390/hematolrep15010022 (PMC10048276; doi:10.3390/hematolrep15010022)
Supplement: Supplementary file 1 [file hematolrep-15-00022-s001.zip › hematolrep-2149437-supplementary.pdf]

Supplementary Table S1: List of genes and exons included in the Next Generation Sequencing panel (TruSight Myeloid Sequencing Panel®).

ABL1 (4-6)	*CEBPA*	*HRAS* (2-3)	*MYD88* (3-5)	*SF3B1* (13-16)
ASXL1 (12)	*CSF3R* (14-17)	*IDH1* (4)	*NOTCH1* (26-28,34)	*SMC1A* (2,11,16-17)
ATRX (8-10, 17-31)	*CUX1*	*IDH2* (4)	*NPM1* (12)	*SMC3* (10,13,19,23,25,28)
BCOR	*DNMT3A*	*IKZF1*	*NRAS* (2,3)	*SRSF2* (14)
BCORL1	*ETV6*	*JAK2* (12,14)	*PDGFRA* (12,14,18)	*STAG2*
BRAF (15)	*EZH2*	*JAK3* (13)	*PHF6*	*TET2* (3-11)
CALR (9)	*FBXW7* (9-11)	*KDM6A*	*PTEN* (5,7)	*TP53* (2-11)
CBL (8-9)	*FLT3* (14-16)	*KIT* (2,8-11,13-17)	*PTPN11* (3,13)	*U2AF1* (2,6)
CBLB (9-10)	*GATA1* (2)	*KRAS* (2,3)	*RAD21*	*WT1* (7,9)
CBLC (9-10)	*GATA2* (2-6)	*MLL* (5-8)	*RUNX1*	*ZRSR2*
CDKN2A	*GNAS* (8-9)	*MPL* (10)	*SETBP1*	
